# Measurement of Horse Allergens Equ c 1 and Equ c 2: A Comparison among Breeds

**DOI:** 10.1159/000525960

**Published:** 2022-09-01

**Authors:** Susanne Victor, Erik Lampa, Anna Rask Andersen, Guro Gafvelin, Hans Grönlund, Lena Elfman

**Affiliations:** ^a^Department of Medical Sciences, Occupational and Environmental Medicine, Uppsala University, Uppsala, Sweden; ^b^Department of Medical Sciences, Clinical Epidemiology, Uppsala University, Uppsala, Sweden; ^c^Department of Clinical Neurosciences, Therapeutic Immune Design Unit, Karolinska Institutet, Stockholm, Sweden

**Keywords:** Dander, Equ c 1, Equ c 2, Horse allergen, Horse breeds, Saliva

## Abstract

**Introduction:**

Horse allergens are less studied than allergens from other furry animals and these allergens must be evaluated to understand the complexity of allergy to horses. The aims of this study were to develop assays for the horse allergens Equ c 1 and Equ c 2 in dander and saliva and to determine their levels in ten horse breeds. The study also included a comparison of these findings with previous results on the levels of Equ c 4 performed on the same study population.

**Method:**

The study population included 170 horses from 10 horse breeds including American Curly and Russian Bashkir horse, which have been suggested to be hypoallergenic. Competitive ELISA assays were developed, with polyclonal antibodies as capture antibodies, for the detection of Equ c 1 and Equ c 2 in dander and saliva samples.

**Results:**

The horse allergens Equ c 1 and Equ c 2 were found in all dander and saliva samples from the ten horse breeds. The GM level (ng/µg protein) of Equ c 1 in dander was 470 (range 129–2,569) and in saliva samples, 40 (range 6–160). The GM level of Equ c 2 in dander was 138 (range 18–1,650) and in saliva samples, 0.8 (range 0.03–17). In dander, there were no significant differences in Equ c 1 and Equ c 2 GM levels between stallions, mares, and geldings.

**Conclusion:**

Our results show high intra- and inter-breed variability. Neither the American Curly horse nor the Russian Bashkir horse, earlier categorized as hypoallergenic breeds, was associated with lower allergen levels of Equ c 1, Equ c 2, or Equ c 4 than the other horse breeds investigated.

## Introduction

*Equus caballus* (domestic horse) continues to play a major role in human history although its use has changed greatly over the years and has included the development of a large number of breeds. Allergy to horse has been reported to affect up to approximately 14%, in a study from the north of Sweden [1], but horse allergy and horse allergens are less studied than allergens from domestic furry animals such as cats and dogs. There is a need to evaluate horse allergens further to understand the complex question of horse allergy and to translate this into clinical practice and treatment modalities [2]. There are five horse allergens, Equ c 1 (lipocalin) [3], Equ c 2 (lipocalin) [4], Equ c 3 (serum albumin) [5], Equ c 4 (latherin), [6] and Equ c 6 (lysozyme) [7], listed in the World Health Organization and International Union of Immunological Society (WHO/IUIS) Allergen Nomenclature Database (www.allergen.org).

Equ c 1 is believed to be the major horse allergen [3, 8]. Up to 76% of horse-allergic patients have been reported to be sensitized to Equ c 1 [9] and sensitization to Equ c 1 has also been associated with severe childhood asthma [10]. Equ c 2 seems to have a lower sensitization prevalence (33%) in horse-sensitized patients, although these data are based on analysis of sera from only a few patients [4, 11]. Both Equ c 1 and Equ c 2 belong to the lipocalin protein family together with many other mammalian allergens such as mouse Mus m 1, rat Rat n 1, dog Can f 1, and cow Bos d 2. Lipocalin allergens sequence identity is usually about 20–30% but can be higher [12].

Equine sports are popular all over the world. In Sweden, it is the second largest sport after soccer and engages more than half a million people. Horses have also shown to be of importance for various therapies, for example, for people with a spectrum of autism [13]. However, many people cannot indulge in horse activities because of allergies, which has stimulated ongoing research to find out if there are any horse breeds better suited for horse-allergic individuals − so called hypoallergenic breeds. Hypoallergenic horse breeds have been suggested, such as the American Curly horse [14], but in a recent study Curly horses were not associated with lower allergen levels in hair and in air samples compared to other breeds [15].

In Sweden, both the American Curly horse and the Russian Bashkir horse have been imported since the 1990s mainly by families with allergy based on the assumption that these breeds were hypoallergenic and could be better tolerated than other horse breeds. It is, however, unlikely that these two breeds descend from the same origin (abcregistry.org).

Previously, the allergen level of Equ c 4 in dander and saliva has been studied in ten horse breeds [16]. No significant differences in allergen levels of Equ c 4 were found between the breeds. Both the American Curly and the Russian Bashkir horse were investigated in the study and did not show significantly lower values of Equ c 4 than the other breeds, with geometric mean (GM) values just below the overall GM value for all horses. Interestingly, stallions showed higher levels of Equ c 4 than mares and geldings [16]. The aim of this study was to classify different horse breeds and to find evidence of the existence of hypoallergenic breeds.

## Material and Method

### Study Population

The study population consisted of a total of 170 horses from 10 horse breeds: American Curly (AC), American Quarter horse (AQ), Gotland pony (G), Icelandic horse (I), North Swedish Horse (N), Russian Bashkir horse (B), Shetland pony (SP), Standardbred (S), Swedish warmblood (SWB), and Thoroughbred (T). The mean age was 10 years (<1–31 years) and the sex distribution was 87 mares, 27 stallions, and 56 geldings. Samples were collected from 144 horses in 2013 and from 108 horses in 2014. A total of 82 horses were sampled both years, see Table 1. The same study population was used in an earlier study to measure levels of Equ c 4 [16].

Ethical approval was not required, according to the Swedish Board of Agriculture (SJVFS 2015:38, chapter 2 § 15). Written informed consent was received from the horse owners.

#### Sample Collection

Dander and saliva samples were collected at the farm where the horses were stabled. The farms were spread over a big area in central Sweden. Horse dander (HD) was collected by grooming the horses and saliva samples were obtained using Salivette® (Sarstedt, Numbrecht, Germany). HD and saliva samples were prepared as previously described [16, 17]. Not all dander and saliva samples collected could be analysed for both Equ c 1 and Equ c 2 due to lack of material. The dander and saliva samples included in the analysis of Equ c 1 and Equ c 2, which were used for statistical calculations, are presented by breed and sex for year 2013 and 2014 in Table 1.

### Production and Purification of Antigens, pAbs, and Biotinylation of Antigens

Antigen production: native Equ c 1 (nEqu c 1) was produced as previously described [8]. Equ c 2 was produced as a recombinant protein (rEqu c 2) [18].

Polyclonal antibodies (pAbs) were raised in rabbits immunized with either nEqu c 1 or rEqu c 2 by Agrisera (Agrisera AB, Vännäs, Sweden). The rabbits were immunized four times (week 1, 5, 9, and 13) and the final bleeding and serum collection were carried out at week 15 after first injection.

Antibody purification from the sera was performed by affinity-chromatography according to the manufacturer's protocol using NHS HiTrap Column, code. 17-0716-01, lot. 10263475 (GE Healthcare) using an ÄKTA purification system (GE Healthcare). The columns were coupled with 2 mg of either nEqu c 1 or rEqu c 2 antigen. Protein concentration was determined using BCA Pierce^TM^ protein assay (Thermo Fisher Scientific, Waltham, MA). The pAb collected was used as capture antibody in the ELISA assay to measure Equ c 1 and Equ c 2 levels in the dander and saliva samples.

Biotinylation of nEqu c 1 and rEqu c 2 was produced according to the manufacturer's instructions (EZ-Link^TM^ Sulfo-NHS-Biotin lot, Thermo Fisher Scientific) and was used as competitive antigens. To remove uncoupled biotin, desalting was performed on a NAP^TM^ 5 column (GE Healthcare) using phosphate-buffered saline (PBS) as buffer, according to the manufacturer's protocol.

### Equ c 1 and Equ c 2 Standards

The assays for both allergen were optimized using nEqu c 1 and rEqu c as standards. Due to lack of sufficient nEqu c 1 and rEqu c 2 for use as standard to analyse all samples, an in-house dander sample was used and set to the concentration of the nEqu c 1 and rEqu c 2 standard curve, respectively. The concentration of the Equ c 1 standard curve ranged from 22.5 to 2,864 ng/mL and the Equ c 2 standard curve ranged from 34 to 2,149 ng/mL. Results were expressed as ng/µg protein.

### Equ c 1 and Equ c 2 Competitive Assay

Ninety-six well Nunc-Immuno MaxiSorp Plates (Thermo Scientific) were coated with Affinity Pure Goat Anti-Rabbit IgG, 5 μg/mL (Jackson Immunoresearch) 100 μL/well, diluted in PBS, and incubated overnight at 4°C. The plates were washed three times with PBS containing 0.02% Tween® (PBS-T) between steps and then blocked for 2 h with 1% bovine serum albumin in PBS-T. pAbs directed to Equ c 1or Equ c 2 in 1% bovine serum albumin PBS-T were added to all wells at 100 μL per well, followed by incubation for 2 h at room temperature. Blank, standard, samples and controls (100 μL/well) were added to the plates and incubated for 2 h at room temperature. Controls were aliquoted from a sample with known concentration, diluted high and low, to confirm that the values were stable throughout the analyses. Prior to adding to the plates, these solutions were mixed 1:1 with biotinylated Equ c 1 (1.6 ng/mL) or Equ c 2 (2.5 ng/mL), respectively. In the next step, 100 μL per well of streptavidin peroxidase (Jackson Immunoresearch, London) diluted 1/4,000 with Guardian^TM^ Peroxidase conjugate stabilizer/Diluent (Thermo Fisher Scientific) was added. In the final step, 100 µL per well of TMB (Sigma Aldrich, Darmstadt, Germany) was added before stopping the reaction with 0.5 M sulphuric acid. Absorbance was measured at 450 nm.

Samples were run in duplicate and at three-fold dilutions to ensure that the allergen concentration fell within the linear section of the standard curve, generally around the third standard point. Blank samples composed of buffer were included and background absorbance was subtracted from the data points. The acceptable coefficient of variation was set at <20%.

### Statistical Analysis

Differences between the breeds were estimated using a linear fixed-effect model. Age, sex, and breed were included as fixed effects, while horse-specific intercepts and by-breed sampling year were included as random effects. Visual inspection of residual plots based on log transformation of the dander and saliva values did not reveal any deviations from the assumptions of constant variance and normality. All confidence intervals reported are simultaneous confidence intervals that account for the multiple comparisons made. All analyses were done using R [19] version 3.3.1 with the Ime4package. Multiplicity-adjusted 95% confidence intervals for the estimated ratios were obtained using the multcomp package [20]. Associations between the repeated measurements were investigated using Pearson correlation coefficients between the log-transformed measurements. Repeated measurements include only the horses sampled in both 2013 and 2014.

## Results

### Equ c 1 and Equ c 2 Levels in HD and Saliva

**Fig. 1:**
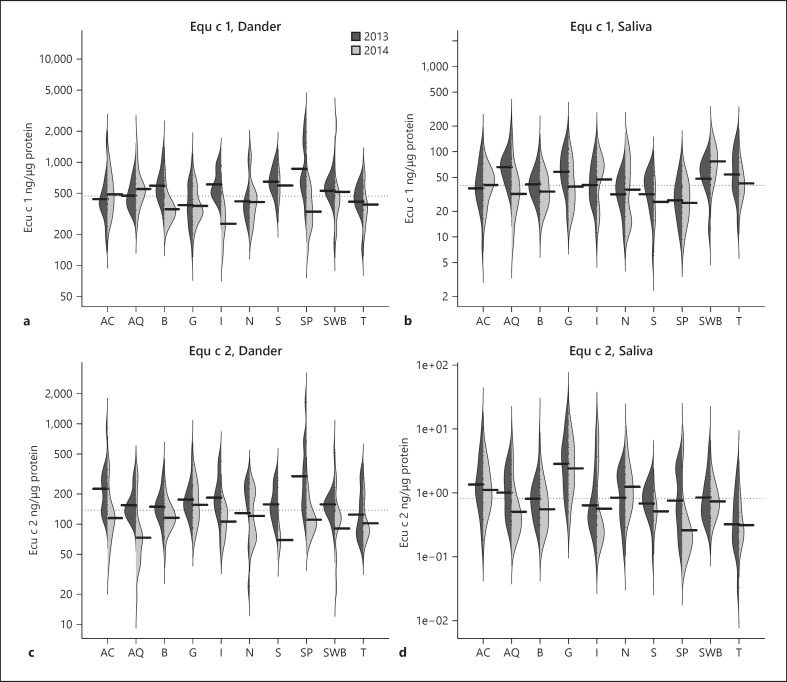
Descriptive figures of the levels of Equ c 1 (ng/µg protein) in dander (**a**) and saliva (**b**) and for Equ c 2 in dander (**c**) and saliva (**d**) for 2013 and 2014 for the ten breeds. The dark grey area represents levels of Equ c 1 and Equ c 2 in samples collected in 2013 and the light grey from 2014. The dotted line shows the overall GM value of all horses, and the black lines show the GM values for each breed and year.

The GM level of Equ c 1 (ng/µg protein) in dander was 470 (range 129–2,569) and in saliva samples, 40 (range 6–160). The GM level of Equ c 2 (ng/µg protein) in dander was 138 (range 18–1,650) and in saliva samples, 0.8 (range 0.03–17). The results are shown in Figure 1.

**Fig. 2:**
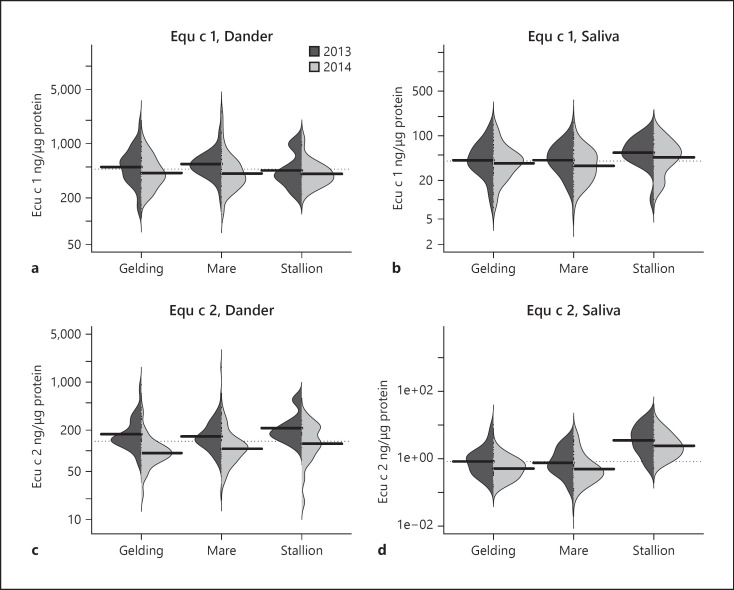
Descriptive figure of the levels of Equ c 1 (ng/µg protein) in dander (**a**) and saliva (**b**) and for Equ c 2 (ng/µg protein) in dander (**c**) and saliva (**d**) by sex and year. The dotted line shows the mean value of all the horses included in the study, and the black lines show the GM values for each sex and year.

The GM levels in saliva are highest for Equ c 2 (Fig. 2d) and slightly higher for Equ c 1 (Fig. 2b) in samples from stallions compared to both mares and geldings in both 2013 and 2014, without any adjustments. Levels of Equ c 1 and Equ c 2 in dander samples were almost equal in mares, geldings, and stallions (Fig. 2).

**Fig. 3:**
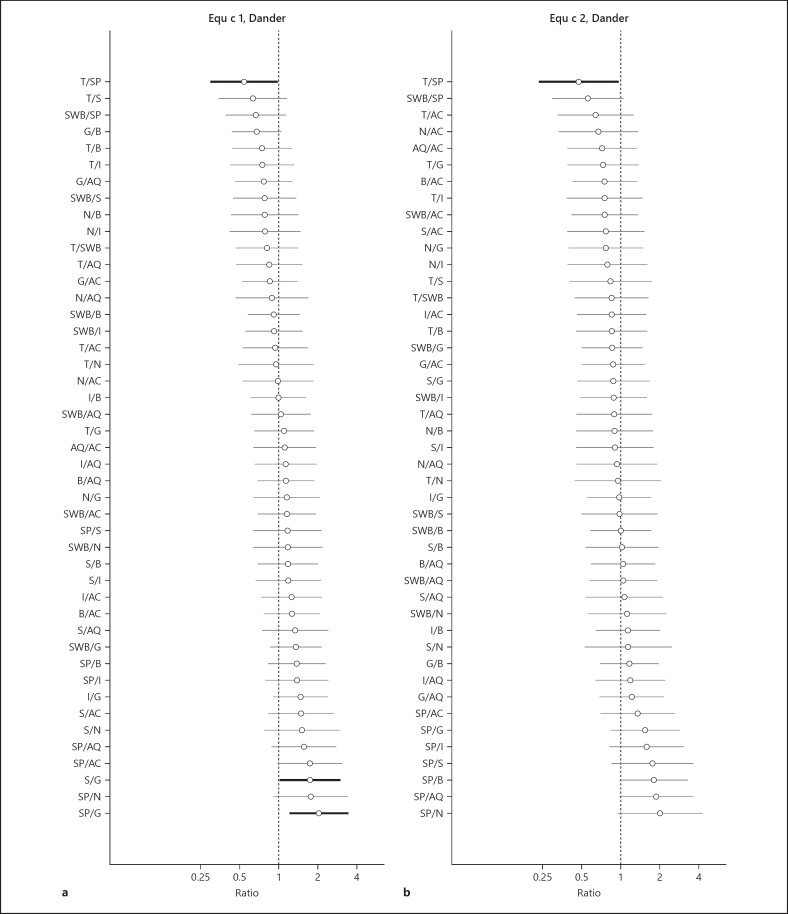
Estimated differences, presented as ratios with 95% confidence intervals, of the breed-specific mean levels in dander for Equ c 1 (ng/µg protein) (**a**) and Equ c 2 (ng/µg protein) (**b**), adjusted for age, sex, and sampling year. Statistically significant differences between breeds are highlighted by a bold line in the figure.

**Fig. 4:**
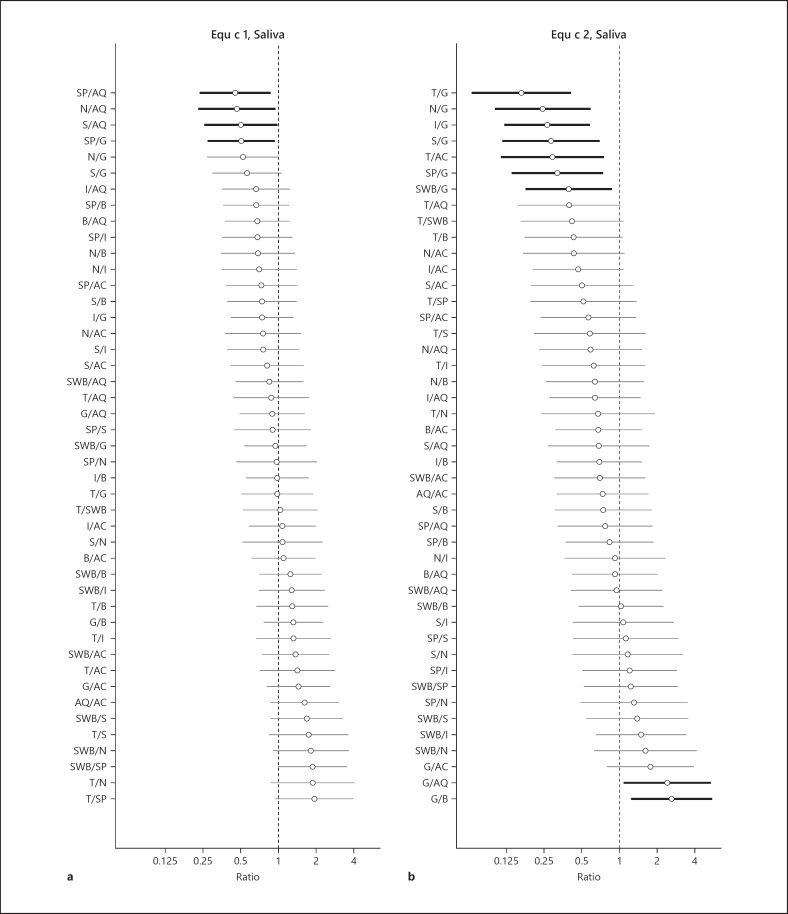
Estimated differences, presented as ratios with 95% confidence intervals, of the breed-specific mean levels in saliva for Equ c 1 (ng/µg protein) (**a**) and Equ c 2 (ng/µg protein) (**b**), adjusted for age, sex, and sampling year. Statistically significant differences between breeds are highlighted by a bold line in the figure.

Comparisons of levels of Equ c 1 and Equ c 2 between breeds, adjusted for age, sex, and sampling year, are presented for dander in Figure 3 and for saliva in Figure 4. The data are presented as increasing ratios where 1 corresponds to equal levels between the compared breeds. For dander samples, the mean level of Equ c 1 for Gotland pony (G) was lower compared to all the other breeds, of which only differences between S and G and SP and G were significant (*p* < 0.05). The mean value for Shetland pony (SP) was higher compared to the other breeds both for Equ c 1 and Equ c 2 but not significantly. The mean level of Equ c 1 and Equ c 2 for SP was significantly higher than Thoroughbred (T) (highlighted in Fig. 3a, b). For the saliva samples, the mean level of Equ c 1 for American Quarter horse (AQ) was higher than for all the other breeds, while significantly higher than SP, N, and S. The mean level for G was significantly higher than for SP (Fig. 4a). The mean values of Equ c 2 for G was significantly higher compared to the other breeds, which is highlighted in Figure 4b.

**Fig. 5:**
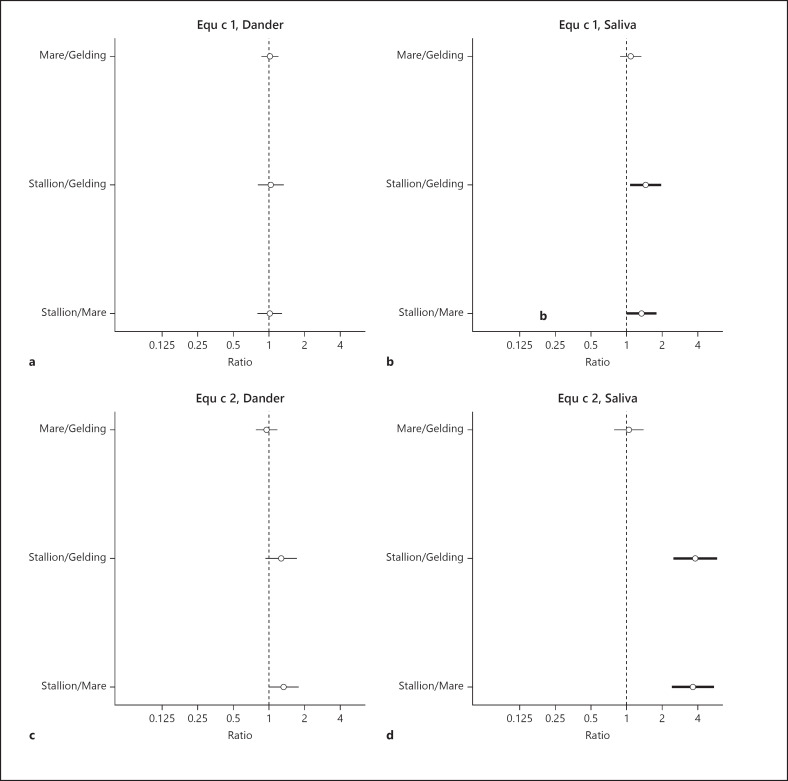
Estimated differences presented as ratios with 95% confidence intervals, of the sex-specific mean levels for Equ c 1 (ng/µg protein) in dander (**a**) and saliva (**b**) and for Equ c 2 (ng/µg protein) in dander (**c**) and saliva (**d**) adjusted for age, breed, and sampling year. Statistically significant differences between sex are highlighted by a bold line in the figure.

Estimated differences in dander and saliva for levels of Equ c 1 and Equ c 2 (ng/µg protein) between stallions, mares, and geldings are shown in Figure 5. In dander, the levels of Equ c 1 were almost the same for stallions, mares, and geldings, but levels of Equ c 2 were slightly higher in stallions. Saliva from stallions showed significantly higher levels of both Equ c 1 and Equ c 2. The GM value of Equ c 2 in saliva was almost four times higher than that of geldings and mares.

To be able to compare horse allergen levels with earlier studies [15], the median levels in dander of all three allergens Equ c 1, Equ c 2 (ng/µg protein), and Equ c 4 (U/µg protein) [16] are presented in Table 2, presenting results from one observation per horse. The median levels were higher in stallions for Equ c 2 and Equ c 4 (178 and 1,847) compared to mares (150 and 612) and geldings (144 and 658), while Equ c 1 in stallions was lower than for geldings and mares.

**Fig. 6:**
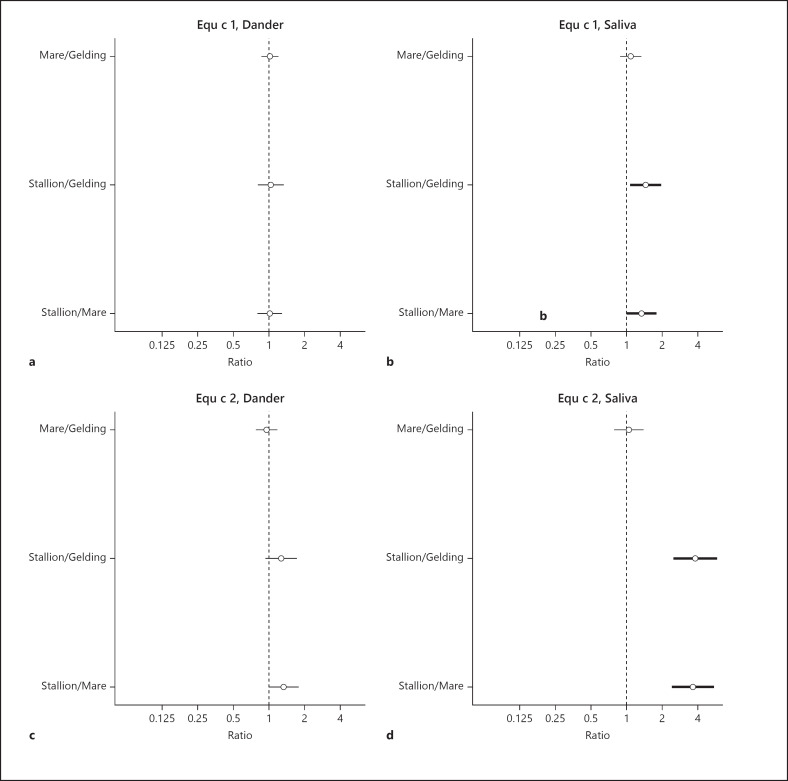
Correlation between levels of Equ c 1 in dander (**a**) and saliva (**b**) and for Equ c 2 in dander (**c**) and saliva (**d**) in samples collected from the same horses in 2013 and 2014.

Allergen levels in dander and saliva samples from horses sampled in both 2013 and 2014 are shown in Figure 6, testing the null hypothesis that the correlation coefficient is equal to zero. The levels correlate poorly to moderate in dander samples for both allergens, Equ c 1 (*p* = 0.477, Fig. 6a), Equ c 2 (*p* = 0.005, Fig. 6c). The corresponding values for saliva samples showed weaker correlation for the horses sampled both in 2013 and 2014, Equ c 1 (*p* = 0.034, Fig. 6b), Equ c 2 (*p* = 0.012, Fig. 6d).

## Discussion

In this study, levels of the horse allergens Equ c 1 and Equ c 2 in ten horse breeds were compared. These allergens were present in dander and saliva samples from all breeds. An earlier study from this research group showed the same result for the allergen Equ c 4 from the same horses [16]. The results showed high intra- and inter-breed variations. No significant differences between breeds were found. In our earlier study, Equ c 4 levels were shown to be highest in stallions both in dander and saliva samples. However, this pattern for sex difference was not as clear for Equ c 1 and Equ c 2 since only Equ c 2 levels in saliva showed significantly higher levels in stallions than mares and geldings when adjusted for age, breed, and sampling year. To our knowledge, this is the first study to present data on levels of the horse allergen Equ c 2.

Similar results with high variability in allergen content among individual animals and breeds have also been shown by Zahradnik et al. [15]. Their study covered 224 hair samples (HD antigen) from 32 horse breeds, of which 11 breeds were represented by only 1 horse and 5 breeds by only 2 horses. They concluded that stallions displayed higher median levels of Equ c 1 and Equ c 4 than mares and geldings. While the data on Equ c 4 agree with our previously published results, here we show similar Equ c 1 levels for all the sexes. Furthermore, their study showed higher concentrations of Equ c 1 and Equ c 4 in American Curly horses compared to most of the horses investigated. In the present study, American Curly horses and Bashkir horses showed similar median levels of Equ c 1, Equ c 2, and Equ c 4 in dander samples compared to median levels of all breeds (Table 2).

From Figure 1, we can conclude that Equ c 1 GM levels for American Curly and Bashkir horse were similar to all breeds (see dotted line in Fig. 1). Equ c 2 GM levels of American Curly were higher in both dander and saliva samples compared to GM levels of all breeds, while the GM values for the Bashkir horse were equal (dander) or slightly lower (saliva) than for other breeds (Fig. 1c, d). From our earlier study, it can be concluded that GM levels of Equ c 4 in dander for American Curly and Bashkir were almost the same as the GM level for all breeds, while the GM level in saliva samples was slightly higher for American Curly and slightly lower for the Bashkir than the GM level for all breeds [16]. Taken together, there is not much evidence for the notion that American Curly and Bashkir horse breeds are hypoallergenic.

Adjusted comparisons between breeds, presented as ratios, show that the mean value for Equ c 1 in dander samples was lower in Gotland pony and higher in Shetland pony compared to all the other breeds. The mean value for Equ c 2 in dander samples was lower in Thoroughbred and higher in Shetland pony than all the other breeds. Data from the earlier study showed that Equ c 4 was higher in the American Quarter horse compared to all the other breeds.

This knowledge may have implications in the future, when component-resolved diagnostics (CRD) may also become available for horse allergens [21]. CRD is beginning to gain greater recognition and clinical utility in fine-tuning the diagnostics of allergy to furry animals [22]. One possibility is that CRD could indicate that a horse-allergic individual may tolerate exposure to certain individual horses but not others.

The strength of this study is that allergen levels in both dander and saliva were studied for a large number of horses: 170 from 10 different horse breeds. Efforts were made to ensure that the samples were representative. A variety of ages and sexes were selected for the sampling. The samples were collected during the summer when the horses were in the fields and not trimmed. In addition, levels of allergens were compared between the same individuals and found to be stable.

The values cannot be regarded as absolute values but rather relative values for comparison between individual horses and breeds for each assay. The assays used for measuring Equ 1 and Equ c 2 used pAbs and a competitive ELISA method, while the Equ c 4 assay was based on commercially produced monoclonal antibodies (Mabtech, Stockholm, Sweden) with an in-house standard [17]. More research is needed to be able to compare the different allergen levels with each other.

To conclude, our results show high intra- and inter-breed variability for the horse allergens Equ c 1, Equ c 2, and Equ c 4. Neither the American Curly horse nor the Bashkir horse was associated with lower allergen levels of Equ c 1, Equ c 2, or Equ c 4 than the other horse breeds investigated.

## Statement of Ethics

Ethical approval was not required, according to the Swedish Board of Agriculture (SJVFS 2015:38, chapter 2 § 15). Written informed consent was received from the horse owners.

## Conflict of Interest Statement

The authors declare no conflicts of interest.

## Funding Sources

This study was supported with grants from the Erling Persson family foundation, the Asthma and Allergy Foundation, the Cancer and Allergy Foundation, and the regional agreement on medical training and clinical research (ALF) between Uppsala County Council and Uppsala University.

## Author Contributions

Susanne Victor involved in conceptualization, data collection and analysis, methodology, project administration, and writing manuscript. Erik Lampa involved in the statistical analysis and interpretation of the data. Anna Rask Andersen involved in writing − review and editing. Guro Gafvelin involved in conceptualization and writing − review and editing. Hans Grönlund involved in conceptualization, methodology, project administration, and supervision. Lena Elfman involved in conceptualization, data collection and analysis, methodology, project administration, writing manuscript, and supervision.

## Data Availability Statement

All data generated and analysed during this study are included in this article. Further enquiries can be directed to the corresponding author Susanne Victor.

## Figures and Tables

**Table 1:** Dander and saliva samples used for calculation

Breed	2013				2014			
	gelding	mare	stallion	total	gelding	mare	stallion	total
AC	**7**	**6**	**1**	**14**	**7**	**5**	**6**	**18**
Equ c 1	5/7	6/6	1/1	12/14	7/7	4/5	5/6	16/18
Equ c 2	5/6	6/6	1/1	12/13	7/7	5/5	6/6	18/18
AQ	**5**	**8**	**0**	**13**	**7**	**10**	**2**	**19**
Equ c 1	4/5	7/8	0/0	11/13	6/7	9/8	2/2	17/17
Equ c 2	4/5	7/8	0/0	11/13	6/7	9/8	2/2	17/17
B	**7**	**12**	**1**	**20**	**7**	**11**	**1**	**19**
Equ c 1	7/7	12/12	1/1	20/20	7/7	11/11	1/1	19/19
Equ c 2	7/7	12/12	1/1	20/20	7/7	11/10	1/1	19/18
G	**9**	**7**	**4**	**20**	**0**	**5**	**5**	**10**
Equ c 1	9/9	7/7	4/4	20/20	0/0	5/4	5/5	10/9
Equ c 2	9/9	7/7	4/4	20/20	0/0	5/4	5/5	10/9
I	**6**	**8**	**2**	**16**	**2**	**3**	**2**	**7**
Equ c 1	4/6	7/8	2/2	13/16	1/2	3/3	2/2	6/7
Equ c 2	4/6	7/8	2/2	13/16	2/2	3/3	2/2	7/7
N	**1**	**4**	**5**	**10**	**1**	**3**	**6**	**10**
Equ c 1	1/1	3/4	3/5	7/10	1/1	3/3	5/6	9/9
Equ c 2	1/1	3/4	3/5	7/10	1/1	3/3	6/5	10/9
S	**4**	**7**	**1**	**12**	**0**	**2**	**0**	**2**
Equ c 1	4/4	6/7	0/1	10/12	0/0	2/2	0/0	2/2
Equ c 2	4/4	6/7	0/1	10/12	0/0	2/2	0/0	2/2
SP	**2**	**11**	**0**	**13**	**1**	**10**	**0**	**11**
Equ c 1	1/2	10/11	0/0	11/13	1/1	10/10	0/0	11/11
Equ c 2	1/2	10/11	0/0	11/13	1/1	10/10	0/0	11/11
SWB	**6**	**8**	**2**	**16**	**4**	**3**	**0**	**7**
Equ c 1	6/6	8/8	2/2	16/16	3/4	3/3	0/0	6/7
Equ c 2	6/6	8/8	2/2	16/16	4/4	3/3	0/0	7/7
T	**4**	**6**	**0**	**10**	**3**	**2**	**0**	**5**
Equ c 1	4/4	6/6	0/0	10/10	3/3	2/2	0/0	5/5
Equ c 2	4/4	6/6	0/0	10/10	3/2	2/2	0/0	5/4
Total	**51**	**77**	**16**	**144**	**32**	**54**	**22**	**108**
Equ c 1	45/51	72/77	13/16	130/144	29/32	52/50	20/22	101/104
Equ c 2	45/50	72/77	13/16	130/143	31/31	53/49	22/22	106/102

The number of sampled horses is presented in bold. Numbers of dander/saliva samples, sorted by breed and sex, analysed for Equ c 1 and Equ c 2, sampled in 2013 and 2014, respectively.

**Table 2:** Median levels of horse allergens

	Nobs	Equ c 1		Equ c 2		Equ c 4	
		median	IQR	median	IQR	median	IQR
Breed							
AC	20	398	329–488	133	115–249	749	360–1,853
B	20	579	464–702	147	121–175	508	312–952
T	10	431	365–551	108	78–177	739	558–1,401
G	24	367	263–595	164	142–237	736	400–1,036
SWB	18	538	472–623	150	126–169	705	567–1,507
I	16	522	499–907	181	138–240	1,020	467–1,956
N	12	386	291–513	183	66–224	453	357–1,685
AQ	24	501	428–600	133	68–177	1,160	641–2,007
SP	14	691	535–1,427	238	143–449	468	329–621
S	12	641	547–780	158	125–214	1,284	553–1,974

Total		512		150		738	

Sex							
Gelding	56	496	344–645	144	123–224	658	406–1,264
Mare	87	535	432–656	150	117–221	612	373–1,175
Stallion	27	410	343–539	178	127–237	1,847	1,146–2,334

Median levels and IQR of horse allergen Equ c 1, Equ c 2 (ng/µg protein), and Equ c 4 (U/µg protein) in HD samples according to breed and sex.
